# New Therapeutic Options for Fusariosis: A Patent Review (2008–2023)

**DOI:** 10.3390/jof11060463

**Published:** 2025-06-18

**Authors:** Izadora Dillis Faccin, Túlio Máximo Salomé, Gleyce Hellen de Almeida de Souza, Leonardo da Costa Xavier, Izabel Almeida Alves, Vanessa Castro Felix Lima, Fabíola Lucini, Simone Simionatto, Luana Rossato

**Affiliations:** 1 Health Sciences Research Laboratory, Universidade Federal of Grande Dourados, 79804-970 Dourados, Brazil; izadora.faccin051@academico.ufgd.edu.br (I.D.F.); tulio.salome116@academico.ufgd.edu.br (T.M.S.); gleycehas@gmail.com (G.H.d.A.d.S.); fabiolalucini10@gmail.com (F.L.); simonesimionatto@ufgd.edu.br (S.S.); 2 Pharmacokinetics and Pharmacometrics Laboratory, Universidade Federal da Bahia, 40110-909 Salvador, Brazil; leocx98@gmail.com (L.d.C.X.); izabelalmeidaalves@gmail.com (I.A.A.); 3 Postgraduate Program in Pharmaceutical Sciences at the State University of Bahia, 41150-000 Salvador, Brazil; vanessa_castrolima@hotmail.com

**Keywords:** *Fusarium*, fungal infection, antifungal agents, drug innovation

## Abstract

Fusariosis is an infection caused by the fungus *Fusarium* spp., which is pathogenic to both plants and humans. The disease presents several clinical manifestations and epidemiological patterns. Current treatment relies on azoles and polyenes, but increasing antifungal resistance requires the exploration of new therapeutic options. This study reviewed patents related to the treatment of Fusariosis from the last 15 years (up to June 2023). The search identified 318 patents, categorized by identification code, publication date, type of application and mechanism of action, using the International Patent Classification and Cooperative Patent Classification systems. In addition, we conducted a bibliographic search in the PubMed database using the same criteria to identify the number of scientific articles. Of the 318 patents, 21 targeted Fusarium infections in humans. The years 2014 and 2018 stood out with three patents each, while the same period recorded an average of 58 published articles. The patents addressed mechanisms such as drug delivery, gene expression, immunotherapy, engineered drugs, and novel compounds. This research highlights the urgent need for continued innovation in therapeutic technologies to effectively treat *Fusarium* wilt.

## 1. Introduction

*Fusarium* infections are a group of serious fungal infections that can affect both plants and humans. In humans, these infections can range from superficial infections, such as keratitis in immunocompetent hosts, to severe disseminated infections, often presenting as fungemia in immunocompromised individuals [1]. Studies have shown that approximately 60% of all human infections caused by *Fusarium* species are attributed to the *Fusarium solani* species complex (FSSC), while the *Fusarium oxysporum* species complex (FOSC) accounts for 20% of these infections. The FSSC includes many opportunistic species (e.g., *F. falciforme*, *F. keratoplasticum*, and *F. petroliphilum*) with high prevalence. However, other *Fusarium* species, such as *F. oxysporum*, *F. verticillioides*, and *F. proliferatum*, are also clinically significant [2]. The incidence and prevalence of *Fusarium* spp. infections vary depending on the underlying disease and geographical region, with rates reaching 20 per 1000 recipients of allogeneic hematopoietic stem cell transplantation with human leukocyte antigen (HLA)-mismatched related donors in Brazil and the USA [3,4].

The treatment of Fusariosis is guided by international recommendations, primarily developed by organizations such as the Infectious Diseases Society of America (IDSA) and the European Confederation of Medical Mycology (ECMM) [5]. According to these guidelines, the first-line treatment for invasive Fusariosis typically involves the use of high-dose liposomal amphotericin B or voriconazole. The choice of antifungal therapy is often influenced by the patient’s immune status, the severity of the infection, and the specific *Fusarium* species involved [6]. In cases of localized infections, such as keratitis, topical agents like natamycin may be preferred. Fusarium infections are a group of serious fungal infections that can affect plants and humans. In humans, these infections can range from superficial infections, such as keratitis in immunocompetent hosts, to severe disseminated infections, often presenting as fungemia in immunocompromised individuals [1]. Two species complexes associated with severe human cases are Fusarium solani, responsible for more than 50% of cases, and Fusarium oxysporum, responsible for 20% of these cases [2]. The incidence and prevalence of Fusarium spp. infections vary depending on the underlying disease and geographic region, with rates reaching 20 per 1000 allogeneic hematopoietic stem cell transplant recipients with human leukocyte antigen (HLA)-mismatched related donors in Brazil and the USA [3,4]. Mortality rates in immunocompromised patients can exceed 80%, especially in those with hematologic malignancies, even when undergoing aggressive antifungal therapies [7,8,9]. Recent studies have shown that *Fusarium* strains isolated from clinical samples often exhibit high Minimum Inhibitory Concentrations (MICs) for commonly used antifungals, such as amphotericin B, azoles, and echinocandins, indicating significant resistance. This resistance complicates treatment and can lead to poor clinical outcomes [10,11]. In addition, *Fusarium* strains isolated from clinical samples have been reported to have high epidemiological cutoff values for common antifungal drugs [12]. The guidelines emphasize the importance of early diagnosis, aggressive treatment, and, where possible, managing underlying immunosuppressive conditions to improve outcomes. Despite these recommendations, the growing incidence of antifungal resistance underscores the need for ongoing research and the development of new therapeutic options [13].

The development of new patents for the treatment of human *Fusarium* infections is important for several reasons. First, it can lead to the creation of more effective treatments than those currently available, which is particularly critical considering the potential severity of these infections and the emergence of drug-resistant *Fusarium* strains [14]. Additionally, new patents may contribute to the development of treatments that are more affordable and accessible to patients worldwide. Many existing treatments for *Fusarium* infections are expensive and may not be readily available in resource-limited settings [1]. Furthermore, new patents can stimulate further research into the causes and mechanisms of *Fusarium* infections, leading to a better understanding of their development and progression. This research may also identify new targets for drug development and other therapeutic strategies [15].

Overall, the significance of new patents for treating human *Fusarium* infections cannot be overstated. They have the potential to significantly improve the quality of life for individuals affected by this challenging fungal infection while also driving further innovation in the field of fungal infection treatment. The aim of this review is to provide a comprehensive overview of potential new drug possibilities for treating human *Fusarium* infections.

## 2. Materials and Methods

### Study Design

This patent review was conducted in accordance with the Preferred Reporting Items for Systematic Reviews and Meta-Analyses (PRISMA) guidelines [16]. The patent search was performed in two public and specialized databases: the United States Patent and Trademark Office (USPTO) and Espacenet of the European Patent Office (EPO). Two independent reviewers (IDF and TMS) extracted the patent data, and in case of disagreements, a third reviewer (LR) was consulted to reach a consensus.

This review includes patents that can be adopted for the treatment of Fusariosis, regardless of whether clinical tests for fungal infections caused by the genus *Fusarium* were performed, as long as they are cited as therapeutic options. The inclusion criteria used were as follows: (1) patents filed in the last fifteen years (up to 2 June 2023), with active or expired status, in any language, containing “Fusariosis and treatment” as keywords in the title or text fields. The exclusion criteria included (1) duplicate and unavailable patents, patents in abandonment or pending publication status, patents published before 2008, and patents outside the scope of the study. The reviewed patents were classified by document identification code (ID), publication date, type of application, mechanism of action, and the International Patent Classification (IPC) and Cooperative Patent Classification (CPC) codes. Additionally, a search of the PubMed literature database was conducted to identify the number of scientific articles published using the same criteria and keywords. All data analyses were performed using the R language [17].

## 3. Results and Discussion

### 3.1. Data Extraction

**Figure 1:**
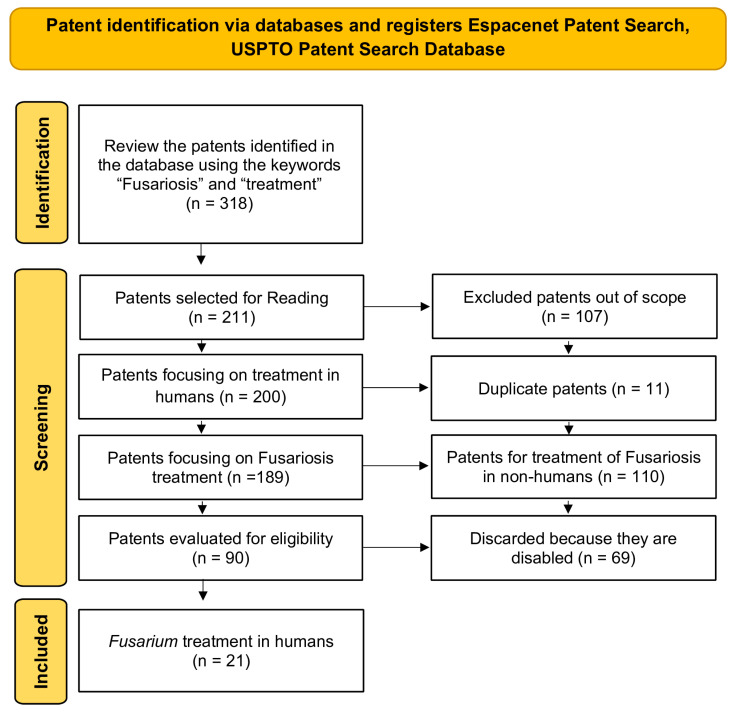
Flowchart of the screening and selection process for patents related to the treatment of Fusariosis (2008–2023), following the PRISMA model.

A total of 318 patents were initially identified from the USPTO and Espacenet databases using the search criteria (Figure 1). After removing duplicates and patents that were outside the time frame or scope of the study, 66 patents were found to describe potential treatments against *Fusarium* spp. Further screenings revealed that only 21 patents met the inclusion criteria for this review, as they specifically described treatments for *Fusarium* infections in humans (Figure 1).

### 3.2. Patent History

**Figure 2:**
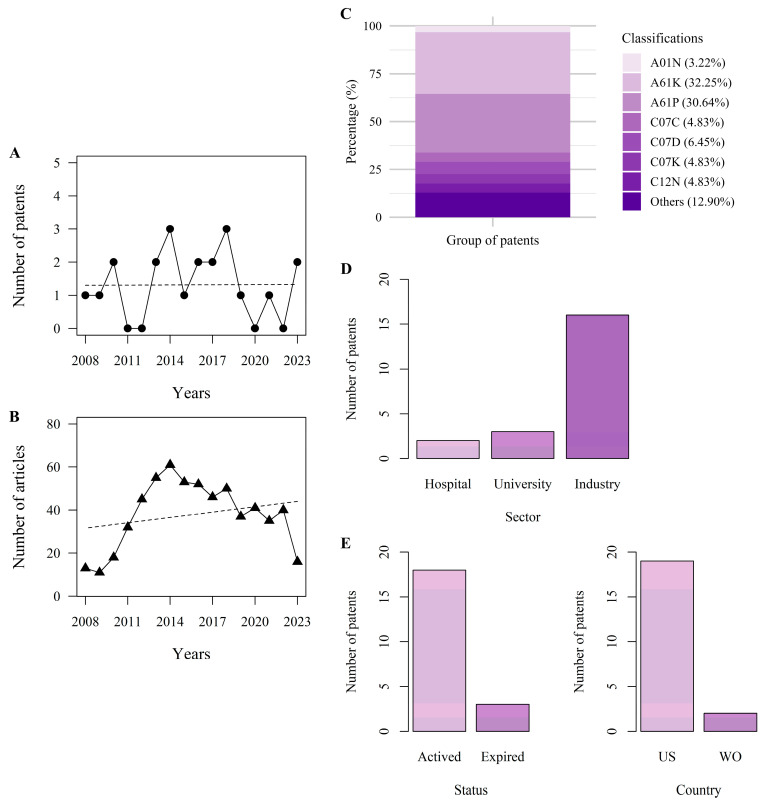
Analysis of patents (**A**) and scientific publications (**B**) related to the treatment of human Fusariosis between 2008 and 2023. Distribution of patent classifications related to *Fusarium* spp. treatment (**C**). Number of patent publications by sector (**D**). Analyzes the distribution of patents by status and country (US: United States; WO: World Organization) (**E**).

No patents were filed in 2011, 2012, 2020, or 2022. On average, 1.4 patents were filed per year during the 15-year period. A total of 21 patents were identified for the treatment of human Fusariosis within this timeframe. The history of patent applications reveals that 2013, 2014, and 2018 had the highest number of filings, with three patents each. The years 2010, 2016, and 2017 had two patents each, while 2008, 2009, 2015, 2019, and 2021 had only one patent each. In contrast, in the first half of 2023, two patents were published (Figure 2A,B).

In addition, a search in the PubMed database using the keywords “Fusariosis and treatment” yielded a total of 605 articles as of 1 June 2023. From 2008 to 2010, the number of publications remained relatively low, ranging from 11 to 18 articles per year. However, a notable increase occurred from 2011 to 2014, with the number of articles rising from 32 to 61 annually.

Furthermore, in 2015, the number of publications reached 53, reflecting a consistent period of activity in Fusariosis research. This pace was maintained in the following years, with 52 articles in 2016, 46 in 2017, and 50 in 2018. Although there was a slight decline between 2019 and 2022, interest in the topic remained constant, highlighting the continued attention to the challenges posed by this infection. As of mid-2023, 16 articles had already been published. Therefore, an average of 37.8 publications per year was achieved over the 15-year period under examination.

These findings shed light on the evolving nature of research activity in the field and provide valuable insights for researchers and stakeholders to assess the state of knowledge dissemination. The data revealed by the analysis of publication and patent trends point to a notable gap between academic research and patent applications in the area of fungal pathologies. Consequently, it is crucial that investment and interest from the pharmaceutical and research industries significantly increase to develop more effective treatments and address this gap, thus ensuring public health and fostering innovation in the treatment of fungal pathologies such as Fusariosis.

### 3.3. Patents per Classification

Patent classification follows the categories described by the International Patent Classification (IPC) and the Cooperative Patent Classification (CPC). The analysis revealed that the majority of patents fell into three main categories: A61K (preparations for medical, dental, or hygienic use), A61P (specific therapeutic activity of chemical compounds or medicinal preparations), and a group labeled as “others” (A61Q, C07B, C07F, C07H, C07J, Y10S, and Y02A). These three categories accounted for 32.25% (20), 30.64% (19), and 12.90% (8) of the patents, respectively (Figure 2C).

The remaining patents were distributed among the following categories: A01N (3.22%, two patents)—linear peptides containing only normal peptide links having 12 to 20 amino acids; C07C (4.83%, three patents)—acyclic or carboxylic compounds; C07D (6.45%, four patents)—heterocyclic compounds; C07K (4.83%, three patents)—fragments of peptides or peptides modified by removal or addition of amino acids; and C12N (4.83%, three patents).

The “other” category includes A61Q—cosmetics or similar toilet preparations; C07B—general methods of organic chemistry; C07F—acyclic, carbocyclic, or heterocyclic compounds containing elements other than carbon, hydrogen, halogen, oxygen, nitrogen, sulfur, selenium, or tellurium; C07H—nucleosides, nucleic acids, sugars, and derivatives thereof; C07J—steroids; Y10S—general markup of cross-cutting technologies spanning various sections of the IPC and technical subjects covered by former USPC cross-referenced art collections; and Y02A—technologies for adapting to the adverse effects of climate change in human, industrial (including agriculture and livestock), and economic activities.

### 3.4. Patent Market Trends

Analysis of patent market trends reveals the prominent role of the industrial sector in patent applications, accounting for 76.2% (16 patents) of the total. This dominance is largely due to strong commercial interests, particularly within the pharmaceutical industry, which invests substantial resources in research and development for drug discovery, indicating its active engagement in innovation and intellectual property protection (Figure 2D). Additionally, universities emerge as significant contributors, responsible for 14.3% (3 patents) of the filings, ranking second. As leading centers for academic study and research, universities play a key role in exploring novel methodologies and therapeutic products.

The absence of patents filed by independent inventors within the identified patents may be attributed to several factors. Independent patent creation and exploitation require substantial technical and scientific support, which can be limited due to inadequate or constrained financial resources and competition with established companies and institutions.

While patenting scientific discoveries or innovative services is crucial for safeguarding intellectual property rights and stimulating investment in research and development, it can also present potential drawbacks. The patenting process often demands prioritizing secrecy and confidentiality, which may hinder scientific collaboration and impede the dissemination of information within the scientific community [18,19].

In the analysis of patent status, a significant majority (18 patents, or 85.71%) of the examined patents are currently held under active ownership, indicating ongoing registration and protection. Only a small portion (3 patents, or 14.28%) have expired and are now in the public domain (Figure 2E). Among the countries, the United States stands out with the highest number of inventions related to *Fusarium* spp. treatment (Figure 2E). According to 2022 indicators published by the World Intellectual Property Organization, the U.S. Patent Office ranked second globally in terms of patent applications [20].

The analysis of IPC classifications has provided valuable insights into the distribution and trends of patent filings across various categories. The dominance of the industrial sector, particularly the pharmaceutical industry, underscores the strong commercial interests driving innovation and research in medical, dental, and hygienic preparations. While patenting plays a critical role in protecting intellectual property rights and incentivizing investment in research and development, it is essential to consider its potential impact on scientific collaboration and knowledge sharing.

### 3.5. Status of the Patents

In this review, we found that the majority of patents related to *Fusarium* spp. treatment are currently active, indicating that the patent holders retain the exclusive rights to use, commercialize, and control the distribution of these inventions. Only a small number of patents have expired, meaning that the exclusive rights granted to the patent holders have ended, allowing these inventions to enter the public domain [21,22]. This patent review provides a comprehensive assessment of potential options and prospective advancements in clinical methodologies and technologies.

However, it is important to note a lack of new or patented products in recent years, particularly in 2011 and 2012. These years coincided with periods of political instability and economic recovery following the 2008 financial crisis in the United States. These circumstances adversely affected production levels in companies, which remained subdued while the unemployment rate stayed high [23]. The global outbreaks of COVID-19 between 2020 and 2022 also required significant knowledge and dedicated technological and scientific resources to contain the spread of the disease [24].

In contrast, the number of articles published between 2008 and 2022 was approximately 22 times higher than the number of patents, indicating that scientific collaboration and ongoing research in the field of *Fusarium* spp. treatment are more prevalent than patenting. This discrepancy can be attributed to the greater bureaucratic burden associated with patent publication, which requires privacy protection and the guarantee of exclusive rights. Additionally, only a portion of the knowledge generated from scientific research is transferred to industry for commercial purposes [25].

### 3.6. Patents Description

The patents discussed here pertain to therapeutic options for fungal infections, with a particular focus on treatment methods targeting fungi of the genus *Fusarium*. Detailed information on these patents can be found in Supplementary Tables S1 and S2.

### 3.7. Drug Delivery

The treatment of *Fusarium* infections is particularly challenging due to the development of intrinsic resistance, both primary and secondary, resulting from exposure to most antifungal agents [8]. Overcoming this limited inhibitory effect presents an opportunity through the utilization of drug delivery systems, especially nanoparticles [26]. The application of nanoparticles aims to enhance the penetration, bioavailability, and pharmacokinetics of antifungal agents, offering a potential solution to combat *Fusarium* infections [27].

The patent application “US 20080248052 A1” describes a novel approach to targeted drug delivery, utilizing a drug delivery conjugate that incorporates a cell surface receptor-ligand along with an optional polyvalent linker [28]. This innovative technology shows great potential in improving the specificity and effectiveness of drug delivery by precisely targeting cells of interest, thereby minimizing side effects. Additionally, it offers the advantage of flexible dosing, allowing for personalized treatment strategies for Fusariosis patients. Overall, this represents a significant advancement in drug delivery with the potential to greatly improve patient outcomes in the treatment of Fusariosis.

The patent application “US 2018098945 A1” presents 184 variations of a nanocarrier structure composed of a silica core enveloped by a lipid membrane containing a charge-retaining agent within its pores [29]. This nanocarrier design is specifically engineered to facilitate the targeted delivery of antifungal agents, including amphotericin B, anidulafungin, caspofungin, fluconazole, flucytosine, isavuconazole, itraconazole, micafungin, posaconazole, and voriconazole. By incorporating a charge-retaining agent within the pores, this invention marks a significant advancement in targeted drug delivery systems. The unique structure and composition of the nanocarrier hold great promise for improving the efficacy and specificity of antifungal treatments.

The patent application “US 2014031366 A1” introduces a pioneering approach for the parenteral administration of azole antifungal agents [30]. The formulations consist of the azole drug dissolved in a first solvent containing an alcohol component (benzyl alcohol or acidified ethanol) and polyethylene glycol. These formulations have demonstrated efficacy in treating Fusariosis. This invention represents an important advance in antifungal therapy, offering a promising option for the parenteral administration of azole agents and expanding the available treatment options. The incorporation of itraconazole into a solution composed of benzyl alcohol, hydrochloric acid, Transcutol P ethanol, and Cyclomethicone has shown promising results for drug delivery and solubilization. The permeability of this formulation exhibited a significant increase of 132% over a 24-hour period, surpassing the performance of the drug in an ethanol solution. Moreover, the concentration of drug deposition in skin and nail tissues achieved levels that were 539% and 230% higher, respectively, compared to the ethanol solution [31]. The development of such optimized drug delivery systems represents a significant advancement in the field, offering improved solubility and efficacy for antifungal agents like itraconazole.

The invention described in “US 9555139 B2” relates to drug delivery conjugates consisting of a linker, a bivalent linker, and tubulysin, along with their analogues and derivatives. These conjugates are designed to enhance targeted drug delivery to specific populations of pathogenic cells. Notably, the conjugates also incorporate additional agents selected for their efficacy against these pathogenic cells, including alkylating agents, protein kinase inhibitors, transcription inhibitors, and others [26]. This patent represents a notable advancement in drug delivery technology and offers promising prospects for the development of innovative treatments in the field of targeted therapy. Tubulysin, a peptide with significant implications in targeted therapy, has emerged as a crucial component in drug delivery strategies [30]. Its mechanism of action involves the disruption of microtubules through antimitotic activity, leading to apoptosis, which has attracted considerable attention in the development of novel drugs [32].

### 3.8. Gene Expression

Gene therapy represents a promising approach to addressing the challenges posed by multidrug-resistant fungi and other diseases [33]. By modulating gene expression in target cells, this therapeutic strategy offers potential solutions to these complex issues. The methodology primarily relies on the use of vectors to introduce genetic modifications into cells in vivo. Notably, the transduction process—where genes are introduced into cells using viral vectors—accounts for a substantial portion (70%) of clinical trials in this field [33,34].

The patent application titled “US 20140274954 A1” introduces boron-containing diacylhydrazine compounds [35]. These compounds contain at least one boron atom in their molecular structure and have the capability to modulate gene expression in host cells. The patent also describes methods for genetically modifying host cells in vivo by administering a viral vector carrying a polynucleotide encoding a switch gene equipped with a ligand-binding domain that specifically interacts with the disclosed compound. This innovation represents a promising avenue for gene therapy and precise control of gene expression. Boron-containing compounds are utilized to modulate the immune system by targeting specific molecular components involved in humoral, innate, and cellular responses. Although these compounds have been primarily employed in treating infectious or neoplastic diseases, the precise mechanisms of modulation are not yet fully understood [36]. This technology offers novel prospects for therapeutic intervention and immune response regulation.

The patent “US 8946294 B2” describes two specific diacylhydrazine compounds: (R)-3,5-dimethyl-benzoic acid N-(1-tert-butyl-butyl)-N′-(2-ethyl-3-methoxy-benzoyl)-hydrazide (compound 1) and (S)-3,5-dimethyl-benzoic acid N-(1-tert-butyl-butyl)-N′-(2-ethyl-3-methoxy-benzoyl)-hydrazide (compound 2). These compounds, which incorporate a diacylhydrazine ligand, play a crucial role in inducible ecdysone receptor-based gene expression systems [37]. They are designed to regulate therapeutic gene expression in host cells, both in vitro and in vivo, with the aim of treating various diseases, including Fusariosis. The development of these diacylhydrazine compounds represents a promising approach to controlling therapeutic gene expression, offering significant potential for advancing gene therapy and its application in treating a wide range of medical conditions.

### 3.9. Immunotherapy

Immunotherapy represents a promising therapeutic approach aimed at enhancing the immune system’s ability to combat *Fusarium* infection. This treatment encompasses three primary types: monoclonal antibodies, immune checkpoint inhibitors, and vaccines. These immunotherapeutic strategies offer significant potential for combating *Fusarium* infection by harnessing the capabilities of the immune system.

The invention described in “WO 2017109028 A1” focuses on the utilization of a recombinant aspartyl protease antigen, specifically the recombinant aspartyl protease Pepl protein from *Cryptococcus* spp., as a treatment of fungal diseases [38]. Additionally, the invention includes protective antibodies that target specific epitopes of the recombinant aspartyl protease antigen, particularly protective anti-Pepl monoclonal antibodies, and their application in treating fungal diseases. This innovative approach enhances the efficacy of vaccines and strengthens the body’s immune response against fungal infections. In another study, gene recombination and subcloning of the aspartyl proteinase 2 protein, a virulence factor of *Candida albicans*, into the pDS56-RBSII-6xhis vector demonstrated effectiveness as a vaccine in protecting mice and promoting the production of IgG and IgA antibodies. This research represents a significant advancement in the fight against Candidiasis and multidrug-resistant infections [39].

The invention described in “US 8444985 B2” presents a novel composition and method for vaccinating individuals against opportunistic fungal diseases, specifically using an inhibitor of a high-affinity iron permease (FTR) polypeptide found in *Rhizopus oryzae* [40]. This innovative approach involves the use of an antibody or antibody fragment that specifically binds to an immunogenic fragment of the FTR polypeptide, providing a targeted and precise method of vaccination. The FTR1 gene is closely associated with the overexpression of high-affinity iron permeases on the cell surface of R. oryzae. These iron permeases play a crucial role in the absorption of iron, which is essential for electron transfer during metabolic reactions and the growth and proliferation of fungi. By inhibiting the function of these iron permeases, the access to iron is reduced, thereby impeding fungal growth [41,42].

### 3.10. Modified Drugs and Association

The patents in this category focus on established pharmacological compounds that have been modified or combined with other compounds to enhance their effectiveness in treating fungal infections, particularly Fusariosis. These modifications and combinations serve various purposes, including reducing side effects, improving bioavailability, enhancing stability and metabolism, increasing efficacy, and optimizing elimination, all aimed at maximizing the compound’s efficiency [43].

The patent “US 8563555 B2” describes a pharmaceutical composition incorporating the Y crystalline form of posaconazole as an active pharmaceutical ingredient [44]. This innovative development allows for the administration of posaconazole in its crystalline form either as a standalone treatment or in combination with other potent pharmaceutically active compounds, including other antifungal agents. By utilizing the distinctive characteristics of the Y crystalline form, these pharmaceutical compositions offer a remarkably effective and versatile therapeutic option for fungal infections. The flexibility to administer the composition independently or in combination with other antifungal agents paves the way for new therapeutic strategies in treating fungal diseases.

Posaconazole is commonly utilized in the form of an oral suspension, where it exists in the S-form, characterized as a hydrated form with approximately three water molecules per drug molecule. However, dehydration leads to its conversion into Form I due to water loss [45]. These findings emphasize the significance of understanding the crystalline form of posaconazole in various formulations and the influence of water on its stability and interconversion between different forms. Such knowledge is crucial for developing pharmaceutical formulations aimed at preserving the Y crystalline form of posaconazole, thereby ensuring its efficacy in treating fungal infections.

The patent “US 2018282291 A1” introduces novel ebsulfur analogues and pharmaceutical compositions containing them [46]. The invention also presents innovative methods for treating fungal infections using ebselen, ebsulfur, and ebsulfur analogues. These compounds offer promising new treatment strategies for fungal infections, with the potential for improved efficacy and flexibility in dosing. Ebselen has demonstrated in vitro activity against *Fusarium* isolates and exhibits synergism with amphotericin B and voriconazole, indicating excellent potential as an antifungal agent for treating Fusariosis [47]. One of the mechanisms of action involves the accumulation of reactive oxygen species (ROS), which may further contribute to the growth-inhibitory effect against fungi [48].

The patent titled “US 9358297 B2” describes an innovative invention concerning aqueous solutions utilized as pharmaceutical compositions of posaconazole [47]. These compositions include a solubilizing agent, such as modified β-cyclodextrin, dissolved in an acidified solution. Additionally, these solutions may contain a chelating agent, such as disodium edetate (EDTA). Initially, posaconazole was exclusively formulated as an oral suspension. However, due to its variable pharmacokinetic profile and the requirement for consumption with a high-fat meal to optimize absorption, alternative formulations have been developed. The intravenous formulation of posaconazole offers an alternative route of administration in cases where enteral absorption is compromised. In this formulation, each 300 mg dose of posaconazole is accompanied by 6680 mg of the solubilizing agent sulfobutylether-β-cyclodextrin [49]. Thus, the aqueous solutions of posaconazole containing a solubilizing agent and a chelating agent provide a promising option for pharmaceutical compositions with improved pharmacokinetic properties for the treatment of Fusariosis.

The patent “US 20100190754 A1” describes the synthesis and evaluation of azolylmethylidenehydrazine and its derivatives for their antifungal activity [50]. The invention revealed that a novel azolylmethylidenehydrazine or its salt exhibited remarkable antifungal activity against various pathogenic fungi responsible for superficial mycosis. Consequently, this invention offers a topical antifungal agent for superficial mycoses in the form of azolylmethylidenehydrazine derivatives. The mechanism of action was investigated in *C. albicans*, where treatment with this compound resulted in increased ROS levels. This elevation in ROS was accompanied by the upregulation of specific genes associated with the anti-oxidative stress response. Moreover, the heightened intracellular ROS induced by this compound caused substantial DNA damage, which played a crucial role in the cellular death triggered by this antifungal treatment [51].

The invention described in “US 20090269380 A1” involves the development of a nanoemulsion-based drug delivery system (oil-in-water) designed to enhance the absorption and efficacy of multiple antifungal agents [52,53]. In vivo studies have confirmed the safety of nanoemulsions for human use. The nanoemulsion droplets are engineered to penetrate skin pores, superficial skin structures, proximal and lateral folds, and nails, effectively reaching the site of fungal infection. These nanoemulsion droplets work by lysing fungal hyphae, cells, and spores, leading to the effective eradication of fungi, molds, and yeasts. This innovation represents a significant advancement in antifungal delivery, enabling efficient penetration of the drug into the skin and nails and resulting in more effective treatment for superficial fungal infections. One specific nanoemulsion, NB-002, has been developed for treating skin, hair, and nail infections. Electron micrographs of fungi treated with NB-002 revealed notable damage to the fungal structure. In laboratory tests, NB-002 demonstrated broad effectiveness against various types of fungi, including *Fusarium* spp., and exhibited rapid fungicidal activity. These promising findings suggest that further research is warranted to explore the potential of NB-002 as a valuable treatment option for cutaneous mycoses [53].

The patents “US 8722727 B2” and “US 2018325919 A1” discuss the compound enfumafungin, which has shown promising inhibitory effects on (1,3)-β-D-glucan synthase, indicating its potential for preventing or treating mycotic infections. Enfumafungin targets (1,3)-β-D-glucan synthase, an enzyme essential for the synthesis of glucan, a critical component of fungal cell walls. Inhibiting this enzyme disrupts fungal cell wall formation, leading to impaired growth and viability of the fungus.

The patent “US 8722727 B2” describes novel compounds, their pharmaceutically acceptable salts, hydrates, and prodrugs, as well as compositions containing these compounds [54]. These compounds have the potential for oral or intravenous administration within a dosage range of 0.001 to 1000 mg/kg body weight per day, either as a single dose or divided doses. A specific example of a dosage range is 0.01 to 500 mg/kg body weight per day, administered orally or intravenously, as a single dose or in divided doses.

On the other hand, “US 2018325919 A1” describes the use of enfumafungin in combination with other mold-active agents such as voriconazole, isavuconazole, posaconazole, itraconazole, and amphotericin B [55]. This patent presents an innovative approach by combining enfumafungin and triterpenoid derivatives with other active antifungal agents for the treatment and prevention of fungal infections, offering a novel and effective strategy against such infections. The orally bioavailable arylamidine enfumafungin (also known as MK-3118, SCY-078, or ibrexafungerp) represents a novel inhibitor of 1,3-β-d-glucan synthesis. It has exhibited remarkable efficacy against multidrug-resistant *C. albicans* and *C. glabrata* isolates [52]. However, its activity against *Fusarium* species is either limited or absent [56]. Nonetheless, a molecular variant of enfumafungin, specifically SCY-078 T-2307, which acts by interfering with cellular metabolism, demonstrates potent in vitro activity (0.125 μg/mL) against *F. solani* [57,58]. These advancements in enfumafungin and its derivatives hold promise for treating fungal infections, and further research is warranted to explore their full potential, including their efficacy, safety, and clinical applications.

The patent “US 11633391 B2” describes the invention related to the use of long half-life 8-aminoquinolines for the treatment or prevention of human lung and respiratory system fungal infections [59]. In some embodiments, the long half-life 8-aminoquinoline is tafenoquine. Tafenoquine is classified as a synthetic 8-aminoquinoline that acts by binding to the DNA of the protozoan or parasite, preventing DNA and RNA production and subsequently inhibiting protein synthesis. Tafenoquine exhibited broad-spectrum activity against medically important yeasts and fungi in vitro and a dose-related antifungal effect in a *Rhizopus* lung infection model at clinically relevant doses [60]. The patent proposes the use of tafenoquine at 2.5 mg/kg and 5 mg/kg as a candidate for preclinical studies for yeast lung infection and COVID-19 co-infection.

### 3.11. New Compounds

Continual research and development efforts are focused on expanding therapeutic options for systemic fungal infections. These efforts involve investigating novel pharmacological compounds designed to target specific diseases and evaluate their effectiveness against infections that may not have been the primary focus of the initial research. These compounds may leverage established mechanisms, whether fully or partially understood, or explore entirely new mechanisms. It is important to note that some of these compounds were originally developed to treat invasive fungal infections caused by other species that share physiological similarities with *Fusarium*. However, translating laboratory discoveries into clinical practice is a lengthy process, and many of these drugs do not reach patients [61].

The patent “US 2019381038 A1” provides valuable insights into the potential application of glucocorticoid receptor antagonists as a therapeutic option for fungal infections [62]. Specifically, PT150 and its derivatives have demonstrated significant antifungal activity by selectively binding to cortisol or cortisol-like receptors present in both humans and pathogens. These receptors play a crucial role in the stress response associated with infections and inflammation. It is essential to emphasize the importance of dosage adjustment, considering factors such as the patient’s age, weight, medical condition, route of administration, treatment schedule, and desired therapeutic outcome. To ensure optimal effectiveness and safety, it is recommended that the maximum daily dose of these glucocorticoid receptor antagonists does not exceed 400 mg/day.

The patent “WO 2021257670 A1” presents an innovative therapeutic approach for fungal infections by administering a compound known as 1A, which specifically targets the glycosylphosphatidylinositol (GPI)-anchored wall transfer protein 1 (GWT1) enzyme in fungi. GWT1 is a conserved inositol acylase involved in the early stages of the GPI-anchored biosynthetic pathway [63]. By inhibiting GWT1, Compound 1A exerts pleiotropic effects on fungal cells, showing great promise in effectively combating fungal infections by targeting essential fungal processes. GWT1 inhibitors are designed to block the transfer activity of a lipid anchor called phosphatidylinositol (PI) to proteins that bind to the cell wall, a process vital for maintaining cellular integrity [64].

The patent “US 9505735 B2” introduces gepinacin, a newly developed compound that specifically targets a critical step in the biosynthesis of GPI anchors in fungi while sparing mammalian cells [65]. This breakthrough has prompted investigations into the impact of inhibiting the biosynthetic pathway on protein homeostasis in fungi and on modifying crucial pathogen-host interactions that contribute to fungal virulence. The compounds described in this patent hold significant value as inhibitors of GPI-anchor biosynthesis, particularly in inhibiting fungal GWT1 activity. Gepinacin’s antimicrobial activity induces overwhelming stress in the endoplasmic reticulum and increases immunogenicity in vitro without causing toxicity in mammalian cell cultures. This is due to the integral role of GPI-anchored proteins in the fungal cell wall structure [66,67]. Therefore, gepinacin holds great promise for potential therapeutic applications in the treatment of fungal infections.

The patent “US 11633434 B2” describes a novel hydrogel formulation comprising a polymer and a microorganism growth medium. The unique aspect of this invention is the incorporation of non-pathogenic viable microorganisms into the hydrogel, effectively inhibiting fungal growth [68]. The hydrogel kit, as described in the patent, includes the hydrogel combined with a specific population of non-pathogenic viable microorganisms. Results in vivo have shown that formulations containing *Bacillus* species within the hydrogel exhibit complete inhibition of *Candida* spp. growth, demonstrating clinical effects comparable to those achieved by ketoconazole [66].

### 3.12. Peptides and Polypeptides

The evolving resistance mechanisms observed in *Fusarium* species necessitate the development of novel antifungal agents that can effectively combat resistance while maintaining a non-toxic profile toward human cells [69]. In response to these challenges, there is growing interest in exploring the potential of antimicrobial peptides (AMPs), particularly antifungal peptides (AFPs), as natural, semisynthetic, or synthetic alternatives [70,71].

AFPs possess unique mechanisms of action, including the disruption of the cell wall and cytoplasmic membrane integrity. This broad-spectrum activity limits the emergence of resistance among pathogenic fungi, providing a significant advantage in combating infections and mycoses [72,73,74]. Consequently, AMPs represent a promising avenue for addressing *Fusarium* infections while potentially overcoming the challenges associated with resistance development.

The invention described in “US 2010184696 A1” is based on the discovery that smaller peptides containing 5 to 15 arginine residues exhibit potent fungicidal properties and hold potential for the treatment of specific fungal infections, particularly Fusariosis [75]. These peptides are hypothesized to penetrate the negatively charged cytoplasmic membrane of the fungus, leading to cell lysis and/or disruption of membrane integrity, ultimately causing the death of the microorganism. The development of these highly fungicidal peptides presents a promising and targeted approach to treating fungal infections, offering a potentially effective solution to combat these pathogens.

## 4. Conclusions

In conclusion, this review underscores the urgent need for sustained investment in the research and development of innovative technologies and therapies to address the challenges posed by Fusariosis, especially in light of the increasing emergence of drug-resistant fungal infections. Future research should prioritize the identification of novel molecular targets and the development of effective therapeutic strategies capable of combating diverse strains of *Fusarium* spp. The creation of new treatments for Fusariosis has significant economic and public health implications, given the considerable morbidity and mortality associated with this infection. Therefore, it is crucial for the scientific community, policymakers, and industry stakeholders to collaborate synergistically to accelerate the advancement and commercialization of novel therapies for this life-threatening disease.

## Data Availability

The datasets generated and/or analyzed during the current study are available from the corresponding author upon reasonable request.

## Supplementary Materials

The following supporting information can be downloaded at: https://www.mdpi.com/article/10.3390/jof11060463/s1, Table S1: Patents for treatments of Fusariosis information; Table S2: Patents chemical compounds.
