# Supplementary material for: New Therapeutic Options for Fusariosis: A Patent Review (2008–2023)
Source: J Fungi (Basel). 2025 Jun 18;11(6):463. doi: 10.3390/jof11060463 (PMC12194352; doi:10.3390/jof11060463)
Supplement: Supplementary file 1 [file jof-11-00463-s001.zip › jof-3217843-supplementary.pdf]

Supplementary Table S1. Patents for treatments of Fusariosis information.

Application number	Formulation administration	Compounds	New technology or/and mechanism of action	IPC
US 20080248052 A1	Oral.	Conjugates of a plurality of cytotoxic drugs and vitamin receptor binding ligands.	Drug delivery.	A61K, A61P.
US 20090269380 A1	Topical.	Nanoemulsion.	Lyse fungal hyphae, cells and spores.	A61K, A61P, Y10S.
US 20100190754 A1	Oral and topical.	Azolylmethylidenehydrazine derivative.	-	A61P, C07D.
US 2010184696 A1	Oral, topical, parenteral, vaginal, rectal, dermal, transdermal, intrathoracic, intrapulmonary and intranasal.	Synthetic peptides.	Charged cytoplasmic membrane.	A61K, A61P, C07K.
US 2014031366 A1	Oral.	Parenteral azole composition with a solvent, lipophilic component and the azole and triazole.	-	A61K.
US 20140274954 A1	Oral.	Boron containing diacylhydrazine compounds.	Regulate gene expression *in vivo*.	A61K, A61P, C07F.
US 2018098945 A1	Oral and topical.	Carriers of porous silica nanoparticles surrounded by a lipid bilayer.	Drug delivery.	A61K, A61P, Y10S.

US 2018282291 A1	Oral, parenteral, intranasal, rectal or topical.	Ebselen, ebsulfur, and ebsulfur analogues.	Reactive oxygen species (ROS) induction.	A01N, A61K, A61P.
US 2018325919 A1	Oral.	Enfumafungin derivative triterpenoid antifungal compounds in combination with other antifungal agents.	Inhibition of glucan synthesis.	A61K, A61P.
US 2019381038 A1	Via oral, nasal, inalatória, tópica, intramuscular, intravenosa, intra-arterial, intraperitoneal, subcutânea	A set of compounds: TPR-1, OR-1, MR-1, TCY1, PT150, PT57, PT158, PT159, PT160, PT162, PT163, PT164, PT165, PT166, PT167.	-	A61K, C07C, C07D, C07H, C07J, A61P.
US 8444985 B2	Oral, topical, suppository and inhaled.	Polypeptide FTR (high affinity iron permease).	Inhibition of iron permease.	A61K, A61P, C12N.
US 8563555 B2	Oral, nasal, transdermal, topical, parenteral and rectal suppositories.	Crystalline form Y of posaconazole.	-	A61K, A61P, Y10T.
US 8722727 B2	Via oral, nasal, parenteral (IV, IM, intraesternal), subcutânea, inalação	Enfumafungin derivatives.	Inhibition of fungal cell wall glucan synthesis.	A61K, A61P, C07D.

US 8946294 B2	Oral.	(R)-3,5-dimethyl-benzoic acid N-(1-tert-butyl-butyl)-N′-(2-ethyl-3-methoxy-benzoyl)-hydrazide (Compound 1) or (S)-3,5-dimethyl-benzoic acid N-(1-tert-butyl-butyl)-N′-(2-ethyl-3-methoxy-benzoyl)-hydrazide (Compound 2).	Regulate gene expression.	A61K, A61P, C07B, C12N, C07C, A01N, Y02A, Y10T.
US 9358297 B2	Intravenous.	Aqueous compositions of posaconazole and a complexing agent.	Improved product life and reduced likelihood of precipitation.	A61K, A61P, Y02A.
US 9505735 B2	Oral, topical, nasal, sublingual intravenous, intramuscular, intraarterial, intramedullary, intrathecal, subcutaneous, intraventricular, transdermal, interdermal, rectal, intravaginal, intraperitoneal, intratracheal instillation, bronchial instillation and/or inhalation.	Compound of formula I.	Inhibits the production of glycosylphosphatidylinositol (GPI) anchors in the endoplasmic reticulum	A61K, A61P, C07C, C07D.
US 9555139 B2	Intravenous and oral.	Conjugates bound to the tubulisin binding ligand.	Drug delivery.	A61K, C07K, A61P
WO 2017109028 A1	Intravenous.	Recombinant antigen of	Pep1 aspartyl protease protein or antibody that	A61K, C07K.

		aspartyl protease.	recognizes specific protective epitopes.	
WO 2021257670 A1	Intravenous and oral.	Compound 1 and Compound 1A.	Inhibits the fungal glycosylphosphatidylinositol (GPI)-anchored wall transfer protein 1 (GWT1) enzyme.	A61K ; A61P
US 11633391 B2	Oral	Tafenoquine and Compound 1	Autoxidation of the 8-amino group, followed by formation of reactive oxygen species	A61K; A61P.
US 11633434 B2	Topical	Hydrogel with non-pathogenic bacteria	Addition of microorganism to generate competition for the microbiota	A61K; A61P; C12N.

Supplementary Table S2. Patents chemical compounds.

Patent	Compound	Chemical Structure
US 20080248052 A1	Conjugates of a plurality of cytotoxic drugs and vitamin receptor binding ligands.	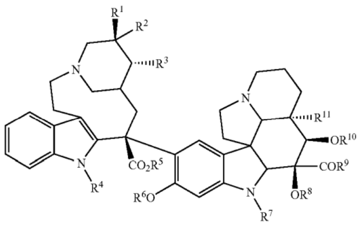
US 20090269380 A1	Nano emulsion containing antifungal.	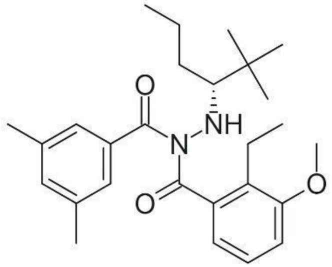 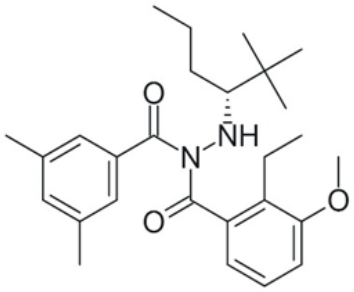

US 20100190754 A1	234 Azolylmethylidenehydrazine derivatives from the general structure.	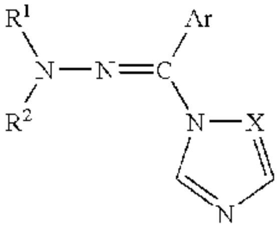 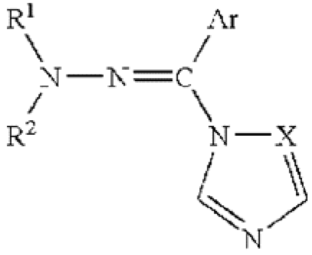
US 2010184696 A1	Synthetic peptides.	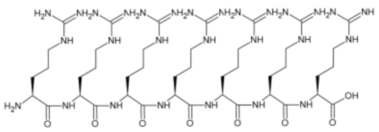

US 2014031366 A1	Parenteral formulations for itraconazole and posaconazole using different solvents.	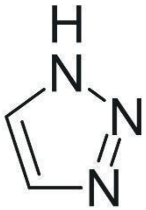 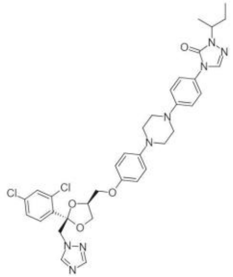 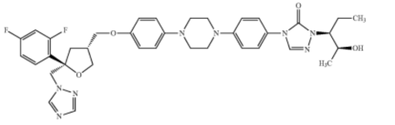
US 20140274954 A1	Boron<br><br>containing diacylhydrazine: (R)-(4-(2-(3,5-dimethylbenzoyl)-2-(2,2- dimethylpentan-3-yl)hydrazine-1-carbonyl)-3-fluorophenyl)boronic acid and N'-(3,5-dimethylbenzoyl)-N'-((R)-2,2- dimethylpentan-3-yl)-7-fluoro-5'-oxo-3H-114 spirobenzoc1,2Oxaborole-1,2'- 1.3.2 oxazaborollidine-6-carbohydrazide.	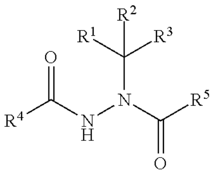 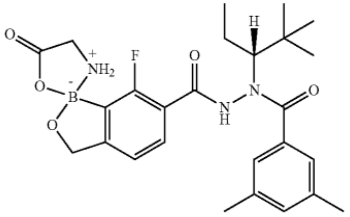

		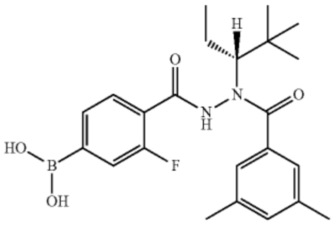
US 2018098945 A1	Silica nanocarrier.	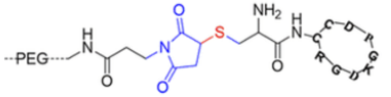
US 2018282291 A1	Ebselen (1-Hydroxy-3,8-naphthalenedisulfonic acid) and Ebsulfur derivatives.	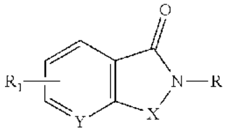 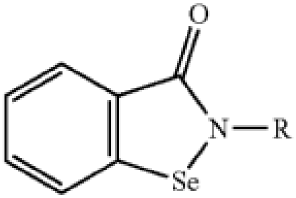 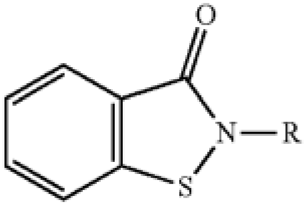

US 2018325919 A1	Enfumafungin (1R,5S,6R,7R,10R,11R,14S,15S,20R,21R)-20-acetyloxy-18-hydroxy-5,7,10,15-tetramethyl-7-[(2R)-3-methylbutan-2-yl]-21-[(2R,3R,4S,5S,6R)-3,4,5-trihydroxy-6-(hydroxymethyl)oxan-2-yl]oxy-17-oxapentacyclo[13.3.3.01,14.02,11.05,10]henicos-2-ene-6-carboxylic acid) and derivate.	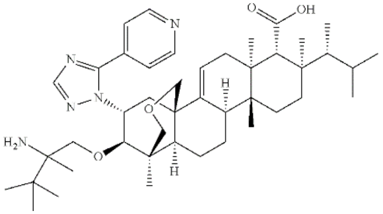 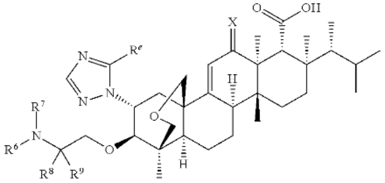
US 2019381038 A1	TPR-1 (No- [3- (Trimethylsilyl) prop - 2 - yn - 1 - yl] rifampi cinium - 4 - olatex3 / 4 HBrxH2O = (2S , 122,14E , 16S , 175,18R , 19R , 20R , 215,22R , 235,24E) -21- (acety loxy) -1,2 - dihydro - 5,6,17,19 - tetrahydroxy - 23 methoxy - 2,4,12,16,18,20,22 - heptamethyl - 8 - [(E) ({ 4 - methyl - 4- [3- (trimethylsilyl) prop - 2 - yn - 1 - yl] piperazin - 4 - ium - 1 - yl } imino) methyl) -1,11 - dioxo - 2,7 (epoxypentadeca [1,11,13] trienimino) naphtho [2,1 - b] furan - 9 - olatex3 / 4 HBrxH2O).	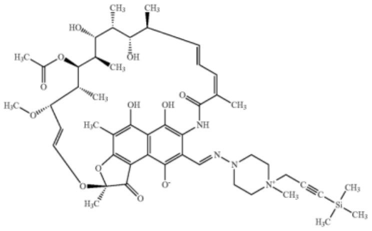
US 8563555 B2	Y-Posaconazole(4-[4-[4-[4-[[(3R,5R)-5-(2,4-difluorophenyl)tetrahydro- 5-(1H-1,2,4-triazol-1-ylmethyl)-3-furanyl]methoxy]phenyl]-1-piperazinyl]phenyl]-2-[(1S,2S)-1-ethyl- 2-hydroxypropyl]-2,4-dihydro-3H-1,2,4-triazol-3-1).	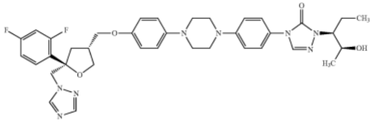
US 8722727 B2	Enfumafungin (1R,5S,6R,7R,10R,11R,14S,15S,20R,21R)-20-acetyloxy-18-hydroxy-5,7,10,15-tetramethyl-7-[(2R)-3-methylbutan-2-yl]-21-[(2R,3R,4S,5S,6R)-3,4,5-trihydroxy-6-(hydroxymethyl)oxan-2-yl]oxy-17-oxapentacyclo[13.3.3.01,14.02,11.05,10]henicos-2-ene-6-carboxylic acid).	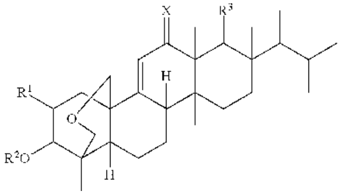 I

US 8946294 B2	Compound 2((R)-3,5-dimethyl-benzoic acid N-(1-tert-butyl-butyl)-N′-(2-ethyl-3-methoxy-benzoyl)-hydrazide (Compound 1) or (S)-3,5-dimethyl-benzoic acid N-(1-tert-butyl-butyl)-N′-(2-ethyl-3-methoxy-benzoyl)-hydrazide).	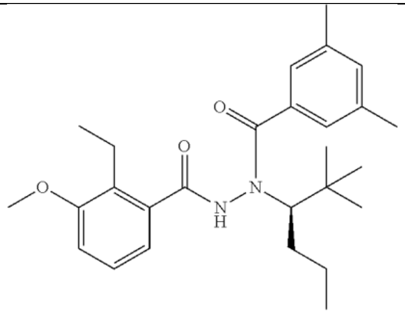
US 9358297 B2	Posaconazole(4-[4-[4-[4-[[(3R,5R)-5-(2,4-difluorophenyl)tetrahydro- 5-(1H-1,2,4-triazol-1-ylmethyl)-3-furanyl]methoxy]phenyl]-1-piperazinyl]phenyl]-2-[(1S,2S)-1-ethyl- 2-hydroxypropyl]-2,4-dihydro-3H-1,2,4-triazol-3-1).	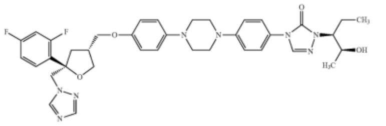 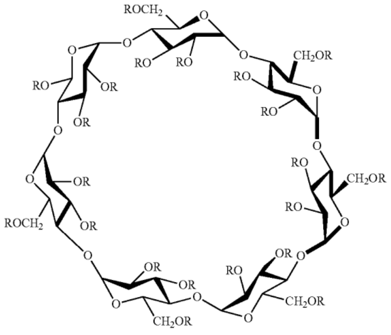
US 9505735 B2	6 compounds derived from Gepinacin (N-(4-methoxyphenyl)-2-[3-(2-methylpropoxy)phenoxy]acetamide2-(3-isobutoxyphenoxy)-N-(4-methoxyphenyl)acetamide).	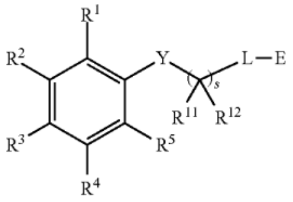

WO 2021257670 A1	Compound 1(2-amino-1-((phosphonooxy)methyl)-3-(3-((4-((2-pyridinyloxy)methyl)phenyl)methyl)-5-isoxazolyl) -pyridinium) and Compound 2(Chemical name not avaible).	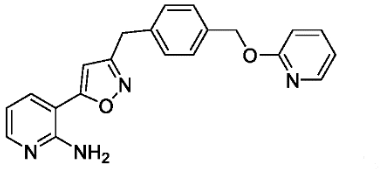 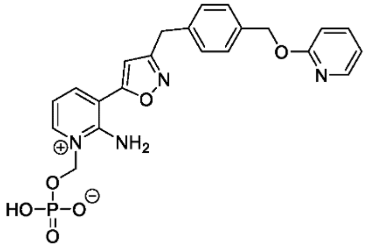
US 11633391 B2	Tafenoquine (8-[(4-amino-1-methylbutyl)amino]-2,6-dimethoxy-4-methyl-5-[3-(trifluoromethyl)phenoxy]quinoline succinate)).	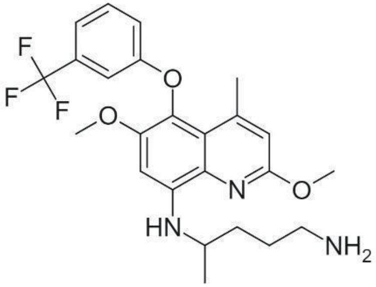
US 9555139 B2	Tubulysin analogues.	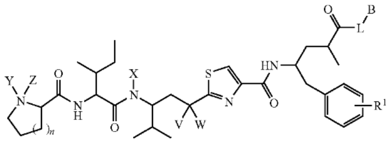
WO 2017109028 A1	a recombinant aspartyl protease protein.	*Not available*
